# Unveiling the Molecular Mechanisms Underlying the Success of Simple Limbal Epithelial Transplantation (SLET)

**DOI:** 10.3390/cells14030200

**Published:** 2025-01-29

**Authors:** Aastha Garg, Kartik Goel, Abha Gour, Mehak Sapra, Virender Singh Sangwan, Ratnakar Tripathi, Anil Tiwari

**Affiliations:** 1 Department of Cornea and Anterior Segment Services, Dr. Shroff’s Charity Eye Hospital, New Delhi 110002, India; aasthagarg93@gmail.com (A.G.); abhagour@gmail.com (A.G.); drsangwan.lvpei@gmail.com (V.S.S.); 2 Department of Stem Cells Research, Dr. Shroff’s Charity Eye Hospital, New Delhi 110002, India; kartikgoel02.1997@gmail.com (K.G.); vohramehak04@gmail.com (M.S.); 3 Department of Veterinary Medicine, University of Missouri, Columbia, MO 65201, USA

**Keywords:** simple limbal epithelial transplantation, limbal stem cell deficiency, cultivated limbal epithelial transplantation, limbal niche, mesenchymal stem cells, corneal transparency

## Abstract

Simple limbal epithelial transplantation (SLET) has emerged as an effective treatment option for limbal stem cell deficiency (LSCD). However, the precise molecular mechanisms underlying its success remain incompletely understood. This review delves into the proposed mechanisms involving the donor limbus, host microenvironment, and the amniotic membrane as a scaffold in SLET. The donor limbus contributes to SLET efficacy through various factors secreted by limbal epithelial stem cells, including hepatocyte growth factor (HGF), soluble Fms-like tyrosine kinase-1 (sFLT-1), and pigment epithelium-derived factor (PEDF), which support corneal healing and transparency. Additionally, the presence of melanocytes, immune cells, limbal fibroblasts, and adhesion molecules within the donor tissue helps preserve the integrity of the limbal niche. The host environment plays a critical role in supporting the transplanted stem cells, with mesenchymal stem cell-secreted factors promoting proliferation and differentiation. Although the amniotic membrane has traditionally been used as a scaffold, emerging evidence suggests that it may not always be necessary. Further studies are needed to validate this scaffold-free approach and to evaluate the vitality and functional contributions of individual components used in SLET. Understanding these complex interactions and molecular mechanisms sheds light on the importance of the donor tissue, host microenvironment, and scaffold in SLET, paving the way for the optimization of this technique for the effective treatment of LSCD.

## 1. Introduction

Limbal stem cells (LSCs) are the adult stem cells of the corneal epithelium. They reside in an anatomically distinct stem cell niche within the limbus. Limbal stem cells play an important role in maintaining the transparency of corneal epithelium. The progenitor cells are located in the anatomical palisades of Vogt and reside in a niche known as the limbal crypts [1]. A major constituent of the limbal niche are the limbal niche cells (LNCs) that regulate the fate of limbal stem cells and maintain the growth of corneal epithelial cells. They have been proven to possess a greater capacity to support the limbal epithelial stem/progenitor cells than limbal stromal cells [2,3,4]. Any pathological condition which disrupts or destroys the normal architecture of the LSC niche or limbal stem cells has the possibility to cause limbal stem cell deficiency (LSCD) [1]. This results in compromise to the barrier function of the limbus and causes the normal corneal epithelium to be replaced with the conjunctival epithelium along with neovascularization and increase in corneal opacity. Thus, conjunctivalization of cornea is a hallmark of LSCD [5]. LSCD is a progressive, ultimately blinding corneal disease of wide-ranging etiology and is found throughout the world [1]. The two main pathological mechanisms of LSCD are the direct loss of limbal stem cells and the loss of their surrounding microenvironment [5]. Chemical injury, aniridia, vernal keratoconjunctivitis (VKC), Stevens–Johnson syndrome/toxic epidermal necrolysis (SJS/TEN), graft-versus-host disease, epidermolysis bullosa, xeroderma pigmentosum are a few common causes for LSCD [1,5].

An insufficient resident population of LSCs to replenish the corneal epithelium and the involvement of visual axis necessitates surgical treatment. Over the years various surgical procedures with which to treat LSCD have been investigated. These have been broadly classified based on the source of stem cells (e.g., autologous, allogeneic); type of stem cell graft (e.g., limbal, oral mucosa, hair follicle, among others); and ex vivo expansion of stem cells in culture [1]. In allogeneic grafts, immunologic rejection remains the main cause of graft failure. These grafts tend to remain at a high risk of rejection and consequent failure even after 3 years of transplantation [1]. The conjunctival limbal autograft (CLAU) is the most commonly performed procedure in cases of unilateral LSCD with a healthy contralateral eye [1]. However, it requires a larger amount of tissue for grafting, which threatens iatrogenic stem cell deficiency [6]. Cultivated limbal epithelial transplant (CLET) was first described in 1997 and addresses the problem by taking a smaller amount of tissue. In CLET, a sheet of cells is expanded for 2 weeks from limbal explant, ex vivo [7]. Simple limbal epithelial transplant (SLET) involves taking a smaller amount of tissue and expanding the cells in vivo on amniotic membrane fixed to the recipient eye. This was first described in 2012 by Sangwan et al. [8]. Cadaveric SLET has been used for a case of bilateral alkali burn [9]. In recent few years, various studies have been performed to test the clinical outcomes and the success of SLET, as summarized in the Table 1.

These studies conclude that autologous and allogenic SLET are all effective, consistent and replicable techniques. SLET helps in corneal regeneration for a longer time period, particularly in unilateral chronic LSCD patients.

**Figure 1:**
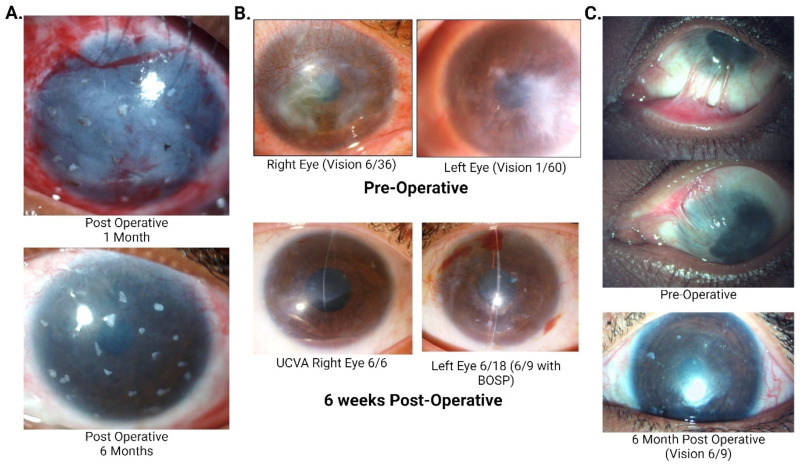
(**A**) Pictures of a 22-year-old male showing progressive corneal clearing over 2 years post simple limbal epithelial transplantation (SLET) after suffering from lime injury with intact limbal biopsies acting as mini-limbus. At a 2-year follow-up, his best corrected visual acuity was 6/18 with contact lens. (**B**) Pre- and post-operative pictures of a 29-year-old female with severe bilateral vernal keratoconjunctivitis (VKC) who underwent bilateral allogeneic simple limbal epithelial transplantation. (**C**) A Six-year-old female child presented with a 2 month history of lime injury.

The anatomical success of SLET in adults (Figure 1A,B) have been reported to be the same as CLET i.e., about 62–80%. In children, (Figure 1C) SLET has been reported to have an even better outcome (73–83%) than CLET (43–45%) [17]. However, CLET is a relatively expensive technique because of the facilities required for cell culture and tissue engineering. It also requires multiple surgeries, one for harvesting and one for seeding, and also increases the probability of cell loss while transferring the cultivated epithelial cells onto the recipient cornea [7].

The molecular mechanisms underlying CLET and SLET are similar; however, the higher success rate observed with SLET may be attributed to its ability to preserve the stromal niche anatomy and integrity in vivo for a longer duration when compared with CLET. This helps in the regeneration and migration of limbal epithelial stem cells for an increased duration of time. SLET involves a complex system of interaction of the epithelial cells with various mesenchymal cells, immune cells, nerves cells, growth factors and cytokines, which have contributed to the remarkable success it has enjoyed for over a decade now. In this communication, we aim to review the probable molecular mechanisms involved in the success of simple limbal epithelial transplantation.

## 2. Method of Literature Search

A primary literature search was conducted in PubMed, MEDLINE, Google Scholar, and Web of Science using the terms simple limbal epithelial translation, cultivated limbal epithelial transplantation, limbal stem cell niche and limbal stem cell deficiency. A secondary literature search was also conducted by reviewing the references of the included articles. All articles up to 31 July 1990 were analyzed. Only English language articles were selected. 

The types of study included the following:

1. Inclusion criteria: randomized control clinical trials, review articles, prospective and retrospective case series, cohort studies, case control studies, animal and laboratory studies.
2. Exclusion criteria: letters, conference abstracts and editorials.

## 3. Proposed Molecular Mechanisms of the Donor Limbal Biopsies

### 3.1. Limbal Niche

Constituents of limbal niche include melanocytes, immune cells like antigen presenting Langerhans cells and suppressor T-lymphocytes, vascular cells, nerve cells and stromal cells like mesenchymal cells [2,3,4]. They closely interact with the limbal basal epithelial/progenitor stem cells with the help of stromal cell-derived factor and its receptor CXCR4 (SDF-1-CXCR4) signaling [18]. Limbal stem cells uniquely interact with extracellular components in the niche by preferentially expressing α9 integrin and N-cadherin [2,3,4]. Limbal niche cells located in the palisades of Vogt are the mesenchymal stem cells residing in the limbal stroma, next to limbal basal epithelial cells. The niche cells provide a protective environment for stem cells from factors that cause differentiation and apoptosis [19]. The limbal basement membrane also helps in sequestering and modulating the concentrations of growth factors and cytokines released by the limbal niche cells for precisely directing the limbal epithelial stem cells [20,21]. The limbal stroma underlying the basement membrane is highly innervated and vascularized [22]. Multiple explants of 0.3–0.5 mm^2^ are created in SLET [23]. These explants act as a “mini-limbus”, over a larger surface area and help in the maintenance of integrity of the entire limbal niche and corneal epithelium generation.

### 3.2. Stem Cells

Polisetty N et al. demonstrated the presence of mesenchymal cells unique to the human limbus, similar to bone-derived MSC (BM-MSC) [24]. Bone marrow-derived mesenchymal stem cells have been studied extensively and it has been shown that they play a definite role in tissue repair. When tissue injury occurs, immune cells release pro-inflammatory mediators and paracrine modulation of the microenvironment is responsible for curbing the tissue damage [25]. Mesenchymal stem cells have direct contact with limbal epithelial stem cells in the niche. They differentiate into keratocytes and elaborate a multilamellar collagenous extra-cellular matrix resembling that of the cornea, thus playing an important role in corneal regeneration and transparency [26,27]. They act by releasing various angiostatic and antifibrotic factors like vascular endothelial growth factor (VEGF), transforming growth factor (TGF)-β, insulin-like growth factor (IGF)-1, hepatocyte growth factor (HGF), fibroblast growth factor (FGF), angiopoietin-1 and stromal cell-derived factor (SDF)-1 which help in tissue repair [25,28]. A brief summary is discussed in Table 2.

#### 3.2.1. Hepatocyte Growth Factor (HGF)

Hepatocyte growth factor is an anti-fibrotic cytokine. It is usually produced by stromal cells in the body [29]. In the cornea, the epithelium, keratocytes, and endothelium produces hepatocyte growth factor [30]. After ocular injury, transforming growth factor beta (TGF-β) is responsible for the development of corneal opacity and scarring due to the differentiation of corneal fibroblasts into α-smooth muscle actin (α-SMA)-expressing myofibroblasts. Mittal et al. have shown that in vitro expanded mesenchymal stem cells, when treated with interleukin-1β (to mimic the inflammatory milieu), enhanced the expression of HGF by 2.5 times and significantly decreased the expression of TGF-β and that that TGF-β1 subsequently induced α-SMA production. The authors speculated that HGF was a putative MSC-expressed factor that could contribute to the restoration of corneal transparency, and demonstrated the same in a mouse model. Here, they knocked down the HGF expression of MSCs using small interfering RNA (siRNA) in mice. Significant corneal opacity was noted post-injury in HGF siRNA-treated mice as compared with controls; as HGF expression was reduced by nearly 80% in the former group [31]. Thus, it can be deduced that the mesenchymal stem cells produced by the limbal stroma secrete high levels of HGF, which inhibits the formation of opacity-inducing myofibroblasts. HGF also acts as an anti-inflammatory cytokine by suppressing TNF-α, monocyte chemoattractant protein-1 (MIP-1), and interleukin-6 (IL-6) expression by a macrophage cell line in vitro and via the antigen-presenting capacity of dendritic cells by downregulating expression of the CD40 co-stimulatory molecule [30]. Animal model studies have also shown that corneal transparency is restored by the topical application of HGF [30,31]. Thus, the preservation of the epithelial–mesenchymal microenvironment in the transplanted biopsies probably helps in recreating the stem cell niche and this may play a key contributing factor in the success of SLET [32].

#### 3.2.2. Soluble Fms-like Tyrosine Kinase-1 (sFLT-1)

Corneal MSCs have been demonstrated to uniquely release soluble fms-like tyrosine kinase-1 (sFLT-1), which is not otherwise secreted by BM-MSC [26]. Eslani et al., in their in vitro and animal models, showed that sFLT-1 acts as an anti-angiogenic factor by causing the sequestration of VEGF ligands, thereby reducing the VEGF-mediated activation of pro-angiogenic receptors and heterodimerization of the full-length VEGF receptor monomers [25]. Thus, it can be assumed that sFLT-1 is an important factor for corneal transparency and is secreted by the limbal stromal cells by inhibiting angiogenesis.

#### 3.2.3. Pigment Epithelium-Derived Factor (PEDF)

PEDF released by MSCs is also considered a potent factor in inhibiting corneal angiogenesis. It acts by various mechanisms. It competitively binds to the VEGFR-2 and causes γ-secretase-mediated cleavage, the translocation of a fragment of the VEGFR-1, and the alteration of the phosphorylation status of VEGFR-l [25,33,34,35].

### 3.3. Keratinocyte Growth Factor (KGF)

KGF-2, a member of the fibroblastic growth factor (FGF-10) family is released by the MSCs and acts by causing the proliferation and regeneration of damaged epithelial tissue [36].

KGF and HGF are expressed differentially by the limbal and corneal fibroblasts and are modulated by cytokines, thereby suggesting their roles in modulating corneal epithelial stem cells and transient amplifying cells [37].

### 3.4. Limbal Fibroblasts

The limbal niche consists of both cellular and extracellular factors. One of the most important components of limbal niche are the stromal fibroblasts. They have an intimate interaction with, and are located in close proximity to, limbal epithelial stem cells (LESCs) in the production of cytokine and growth factors. Limbal fibroblasts (LFs) exhibit mesenchymal stem cell (MSC) characteristics that promote limbal epithelial proliferation, differentiation and limbal epithelium phenotype maintenance during wound healing. These fibroblasts cause a release of several cytokines, including HGF, keratinocyte growth factor and IL-6 [38,39]. A study by Kruse and colleagues has confirmed the importance of limbal stem cell niche fibroblasts to regulate the behavior and differentiation of limbal stem cells into corneal epithelial cells. In this study, they transdifferentiated the hair follicle stem cells into corneal epithelial cells by using a specific extracellular matrix and a fibroblast-conditioned medium that had been isolated from donor corneas which were not suitable for transplantation [40]. The specificity of limbal fibroblasts has also been demonstrated by Amirjamshidi H and colleagues, who used skin fibroblasts to study the growth of corneal epithelium compared with limbal fibroblasts. They found that no growth of corneal epithelium was noted in the mice treated with skin fibroblasts [38]. It has been concluded that the limbal fibroblasts, as maintained in the limbal niche, play a significant role in the reprogramming and formation of highly differentiated epithelial sheets as compared with the MSCs due to the presence of stage-specific embryonic antigen-4 (SSEA4) [39].

### 3.5. Melanocytes

Melanocytes are mainly found in the limbal area, closer to the corneal periphery, and constitute an important part of the limbal niche. They have a myriad of functions like protection of stem cells from UV radiation, free radical scavenging, and immunological support. Melanin-containing epithelial cells align the basal layer of the limbus. The presence of apical pigmentation has been confirmed in K19-positive cells, a cytokeratin expressed exclusively by the limbal basal cells [41]. Melanocytes have direct contact with limbal epithelial stem cells via cadherins and L1-CAM that help in their cellular recognition, binding, and adhesion process [26,42,43]. Sangwan and colleagues have reported the appearance of pigmentation at the involved limbus at 8–12 months post-SLET, which remained stable thereafter. They hypothesized that the transplanted biopsies promote wound healing or that this wound healing might promote melanocyte migration at the limbus. They have suggested that, because the niche is not preserved in ex vivo cultured limbal epithelial grafts, the movement of melanocytes has not been noted in CLET yet [32]. Dziasko MA et al. studied the effect of limbal melanocytes in 2D and 3D cultures and suggested the role of melanocytes present in limbal crypts in maintaining the limbal niche. Their in vitro model showed that limbal epithelial cells grown on mitotically active limbal melanocytes generated large clusters of compact epithelial colonies. In 3D co-cultures, melanocytes were found to cause the promotion of epithelial sheet layers and maintain the basal layers in an undifferentiated state [44]. From the above studies, it can be deduced that the limbal melanocytes play a functional role in initiating and promoting the stratification of limbal epithelial cells in an undifferentiated state due to the preserved limbal niche.

### 3.6. Immune Cells

Mast cells act as innate immune sentinel gatekeepers in the central nervous system, gut, skin, and ocular surface. The presence of mast cells near the palisades of Vogt necessitates the need to understand their contribution close to niches. Studies show that the morphology of these mast cells can appear to be either intact (resting) or degranulated (active), suggesting their dynamic contribution to maintaining the niche [45]. Tryptase expression by mast cells is responsible for regulating cell trafficking and maintaining the local homeostasis of the niche. Mast cell recruitment at stem cell niches by a non-IgE-mediated route (NGF, TGFβ1, and stem cell factor) helps in maintaining homeostasis, protection, and nourishment of the limbal niche [45]. Mast cells have been shown to promote mesenchymal stem cell proliferation/migration as well as inhibit their differentiation into myofibroblasts (myoFBs) in cardiac tissue; as a result, they have a positive role in myocardial infarction [46]. This can be extrapolated to the fact that the presence of mast cells in the limbal niche may prevent the myofibroblast formation, thus contributing towards corneal transparency.

### 3.7. Adhesion Molecules

Cell–cell and cell–matrix adhesion within stem cell niches is crucial for the creation and preservation of niche architecture, the propagation and interaction of governing signals, and the provision of cell polarity cues for control of cell divisions [47]. The anchoring of LESC/LEPCs to their supporting cells and matrix components helps in maintaining a stable stem cell population, thereby allowing constant exposure to niche-related signals. Direct contact between LEPCs and stromal niche supports clonal growth and maintenance of stemness [48,49]. Limbus-specific extracellular matrix and basement membrane composition include laminin α1, α2, β1, and γ3 chains, agrin, tenascin-C, osteonectin/SPARC, and vitronectin in association with LESC clusters [50,51]. Integrins like α6, α9, β1, β4, αvβ5, and N-cadherin, have also been preferentially expressed in the LEPCs of the human limbus [48,52,53].

## 4. Proposed Molecular Mechanisms by the Host

Various factors are upregulated in the corneal epithelium after mechanical or chemical damage to the ocular surface. A significant group of genes responsible for growth and differentiation include transforming growth factor-α (TGF-α), TGF-β, epidermal growth factor (EGF), and fibroblast growth factor-β (FGF-β), which are produced by corneal epithelial cells and which modulate the proliferation as well as the growth of cells on the ocular surface [54]. Insulin-like growth factor-I (IGF-I) has been shown to support the proliferation of keratinocytes, enhance the production of the adherent–junction protein N-cadherin, stimulate the formation of the extracellular matrix, and increase the synthesis of collagen by keratinocytes [55].

### 4.1. Insulin-like Growth Factor (IGF)

IGF-I and IGF-II have been shown to support the proliferation of keratinocytes [56] and the C-domain of both causes the growth of rabbit corneal epithelial cells in synergy with substance P [57]. IGF is rapidly produced by corneal epithelial cells post-injury and is thought to be responsible for the terminal differentiation of limbal stem cells. This was established by Trosan P et al., who cultured limbal cells in the presence of factors produced by the damaged corneal epithelium. Among the various factors, IGF-I was the only factor that induced the expression of the K12 gene. They demonstrated that, post-injury, there is a downregulation of IGF receptors in the corneal cells, which increases the penetration of IGF into the limbal region, stimulating the expression of IGF receptors and limbal stem cell differentiation with no effect on epithelial proliferation [54]. The concentration of epidermal growth factor and fibroblastic growth factor-β were also significantly increased in their model post epithelial injury but these cells did not show the expression of K12 [54].

### 4.2. Risk Factors Contributing to SLET Failure

Various risk factors have been identified for the failure of SLET. These include severe ocular surface damage, such as acid injuries and extensive corneal neovascularization, which can hinder the regeneration of healthy epithelium. Chronic inflammation, often associated with conditions like SJS or OCP, creates a hostile environment that compromises graft survival. Additionally, a prior history of multiple penetrating keratoplasties or extensive ocular reconstruction surgeries poses significant challenges due to altered corneal architecture and stromal integrity. Severe symblepharon further exacerbates the risk of failure by mechanically obstructing the spread and proliferation of transplanted limbal epithelial cells. Addressing these factors is critical to improving the success rate of SLET.

## 5. Biological Scaffold for SLET

### 5.1. Amniotic Membrane

In simple limbal epithelial transplantation, limbal biopsies are placed on a cryopreserved amniotic membrane. The preservation of the amniotic membrane causes loss in immunogenic function along with the loss of viable and active cells [58]. The role of devitalized amniotic membrane in expanding the stem cell niche has been described by Tseng and colleagues. Gap junction molecule connexin 43 is usually not expressed in the limbal basal epithelium but is typically expressed in the corneal basal epithelium. They have shown that connexin 43 expression was reduced on intact amniotic membranes when compared with EDTA-treated amniotic membranes. They conclude that the devitalized epithelium of the preserved intact amniotic membrane maintained a less differentiated epithelial phenotype, similar to the limbal basal epithelium, thereby retaining the characteristics of limbal basal epithelial cells in vivo [59]. The intact amniotic membrane has also been shown to express higher levels of EGF, KGF, HGF, and bFGF when compared with the epithelium-denuded amniotic membrane, which indicates the presence of these growth factors in the amniotic epithelium [60]. The basement membrane of the amniotic membrane also facilitates the adhesion and migration of corneal basal epithelial cells. The stroma contains various anti-angiogenic and anti-inflammatory factors, of which nerve growth factor (NGF) plays a key role in epithelium integrity and stem cell survival. NGF mainly acts through tyrosine kinase-transducing receptor A (TrkA) receptors. A study by Tseng S et al. reports the strong expression of TrkA in the limbal epithelial cells and their preserved phenotype on both intact and epithelial-denuded amniotic membranes after xenotransplant. Higher levels of NGF were noted in the denuded amniotic membrane than in intact AM, indicating the presence of NGF in the stroma [61]. Thus, it would be prudent to conclude that the amniotic membrane serves as an ideal biological scaffold for the implantation, growth, and expansion of limbal explants in vivo.

### 5.2. Fibrin Glue

The amniotic membrane and the limbal biopsies on it adhere to the ocular surface with the use of fibrin glue. This imitates the final stages of the coagulation cascade, where the activated factor X hydrolyses prothrombin to thrombin and where fibrinogen is converted to fibrin in its presence. Thrombin also activates factor XIII in the presence of calcium ions. This stabilizes the clot, thus causing polymerization and cross-linking of the fibrin strands. This causes the subsequent proliferation of fibroblasts and granulation tissue formation [62,63]. Thus, it plays a vital role in limbal epithelial transplantation.

## 6. Recent Advances, Modifications and Future Implications of SLET

### 6.1. SLET Without Scaffold

A recent study by Jain N et al. [64] has described a method for performing simple limbal epithelial transplantation (SLET) without amniotic membrane grafting (AMG) in six patients with limbal stem cell deficiency (LSCD). Limbal explants were placed on the bare cornea and secured with fibrin glue, followed by application of a bandage contact lens. Complete epithelialization occurred within 2–3 weeks, and at a mean follow-up of 9 months, all patients showed stable outcomes with no explant loss. Compared with traditional SLET using AMG, this technique simplifies the procedure by placing limbal explants directly on the corneal surface, reducing costs and avoiding complications associated with AMG, such as infections, dislodgement, and haze. While both methods show comparable outcomes in terms of epithelialization and visual improvement, the absence of AMG may limit the modified approach’s applicability in severe LSCD or cases with irregular corneal surfaces, where AMG provides structural support. The study’s sample size was small with a short follow-up period; however, this simplified technique holds promise, particularly in resource-limited settings, and warrants further investigation to establish its broader clinical utility and safety.

### 6.2. Mini-Simple Limbal Epithelial Transplantation (Mini-SLET) Technique

The mini-simple limbal epithelial transplantation (Mini-SLET) technique it is a promising alternative for managing partial limbal stem cell deficiency (LSCD) in pediatric patients. This approach involves placing the explants directly on the raw corneal surface and covering them with AM. The rate of limbal explant dislocation or displacement in children ranges from 5% to 7%. Children are more prone to epithelial cell growth on contact lenses and are at a higher risk of losing bandage contact lenses (BCLs) during outdoor activities. By improving explant stability, the technique reduces the risk of reoperation, a significant benefit given the additional risks and costs of general anesthesia. Nonetheless, this simplified and innovative approach holds the potential to enhance LSCD management in pediatric populations, especially in developing countries with limited resources [65].

### 6.3. Glueless-Simple Limbal Epithelial Transplantation (G-SLET)

Glueless-simple limbal epithelial transplantation (G-SLET) is a novel modification that addresses the challenges of fibrin glue unavailability and explant loss. By creating radial incisions and tunnels in the corneal periphery for the direct placement of limbal explants, G-SLET eliminates the need for fibrin glue, relying instead on the mechanical stability provided by the tunnels and amniotic membrane suturing [66]. The reported cases [67] have demonstrated the successful and complete epithelialization of 9 of 11 cases (81.8%), partial epithelization in 2 of 11 cases (18.20%) and improved visual acuity in 7 of 11 cases (63.6%), with stable explant positioning over a 6-month follow-up. G-SLET offers significant advantages, including reduced costs and applicability in resource-constrained settings, making it a viable option for managing unilateral limbal stem cell deficiency (LSCD). While the outcomes were positive in the conducted studies, partial success due to preexisting comorbidities highlights the need for patient-specific considerations. This modification holds promise for expanding the accessibility of SLET in settings where fibrin glue is unavailable, though further studies with larger cohorts and longer follow-ups are needed to validate its broader efficacy and safety.

Recently, a modified FSL-assisted G-SLET approach [68] was reported using femtosecond laser (FSL) technology to create precise corneal tunnels. This method was assessed in five porcine eye models and three clinical cases of LSCD caused by chemical burns. Stable corneal epithelialization occurred within 2–3 weeks post-surgery, with healthy epithelium and visible micrografts noted at six months. Best-corrected visual acuity improved significantly in two cases but was limited in one due to severe stromal scarring. Patients reported reduced symptoms and improved quality of life. The FSL-assisted G-SLET technique enhances surgical precision by standardizing corneal tunnel dimensions, improving safety, and demonstrating potential as an effective treatment for LSCD.

## 7. Conclusions

**Figure 2:**
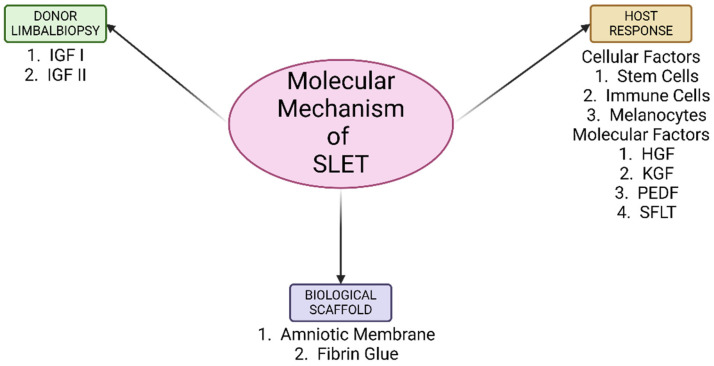
Schematic diagram representing a summary of the molecular mechanism of SLET.

SLET has revolutionized the treatment of LSCD, offering a minimally invasive and cost-effective alternative to other transplantation techniques. This review highlights the intricate molecular mechanisms that contribute to the success of SLET, including the role of donor limbus-secreted factors (Figure 2) such as HGF, sFLT-1, and PEDF, as well as the contributions of melanocytes, immune cells, limbal fibroblasts, and adhesion molecules in preserving the limbal niche. The host microenvironment plays an equally critical role, with mesenchymal stem cell-secreted factors supporting stem cell proliferation and differentiation. Although the amniotic membrane has traditionally been used as a scaffold in SLET to provide structural support and a bioactive surface for transplanted limbal niche, emerging evidence suggests that it may not always be necessary. However, further studies are required to evaluate alternative scaffolds or scaffold-free approaches and their long-term efficacy in clinical settings. Understanding these molecular mechanisms underscores the importance of the dynamic interplay between donor tissue, host environment, and scaffold in ensuring the success of SLET. These insights pave the way for optimizing this technique and potentially enhancing its therapeutic applications. Future research aimed at elucidating additional molecular pathways and refining the procedure could further expand its clinical utility, ultimately improving outcomes for patients with LSCD.

## Figures and Tables

**Table 1:** Summary of studies evaluating the clinical outcomes and success of simple limbal epithelial transplantation (SLET).

Authors	Study Title	Sample Size	Outcomes of SLET
Basu et al. [10]	Long-Term Clinical Outcomes in 125 Cases of Unilateral Chronic Ocular Surface Burns	One hundred twenty-five eyes	Two-line improvement in visual acuity was seen in 75.2% of eyes over a mean follow-up of 35.5 months. A proportion of 67% of the successful cases attained a visual acuity of ≥20/60 (*p* < 0.0001). Progressive conjunctivalization occurred in 18.4% of eyes.
Vazirani et al. [11]	Autologous Simple Limbal Epithelial Transplantation for Unilateral Limbal Stem Cell Deficiency: Multicentre Results	Sixty-eight eyes underwent autologous SLET across eight centres in three countries.	Success rate was 83.8% over a mean follow up of 12 months. A proportion of 64.7% eyes achieved a visual acuity of ≥20/200. A proportion of 64.7% gained a visual acuity of ≥2 Snellen lines. Focal recurrences of pannus were noted in 36.8% cases with clinical success.
Gupta et al. [12]	Results of Simple Limbal Epithelial Transplantation in Unilateral Ocular Surface Burn	Thirty eyes (eighteen adults and twelve children)	Visual acuity gain (≥20/200) was reported in 71.4% of successful cases over a mean follow up of 1.1 years.
Prabhasawat et al. [13]	Efficacy and Outcome of Simple Limbal Epithelial Transplantation for Limbal Stem Cell Deficiency Verified by Epithelial Phenotypes Integrated with Clinical Evaluation	Twenty-eight eyes of twenty-six patients (eleven autologous SLET and seventeen living-related allogenic LSET)	The survival rate was 89.3% at 2 years and 75.6% at 3 years, with statistically significant improvement in visual acuity. No statistically significant difference was noted between autoSLET and Lr-alloSLET.
Wang et al. [14]	Clinical Outcomes of Modified Simple Limbal Epithelial Transplantation for Limbal Stem Cell Deficiency in Chinese Population: A Retrospective Case Series	Thirteen eyes with LSCD. Ten eyes underwent autologous SLET. Three eyes underwent allogeneic modified SLET.	At an approximate 6–7 months follow-up, 77% of the eyes maintained a successful outcome. Two-line improvement was noted in 60% of the cases.
Iyer et al. [15]	Outcome of Allo Simple Limbal Epithelial Transplantation (alloSLET) in the Early Stage of Ocular Chemical Injury	Eighteen eyes of seventeen patients with ocular chemical injury.	Corneal phenotype with complete epithelialisation was achieved in the immediate postoperative period in 17 of the 18 eyes (94.11%) i.e., 22.5 ± 9.14 days.
Shanbhag SS et al. [16]	Simple Limbal Epithelial Transplantation (SLET): Review of Indications, Surgical Technique, Mechanism, Outcomes, Limitations, and Impact	Thirty eyes with LSCD. Sixteen eyes underwent living-related allogeneic SLET. Fourteen eyes underwent cadaveric SLET	The overall success of allogeneic SLET was 83.3%. Successful outcomes at 1-year post-op were maintained in 87.5% of eyes in the living-related SLET group and 78.6% of eyes in the cadaveric group. Kaplan–Meier survival analysis: 5-year cumulative survival probability for 90± 4% of eyes in the living-related group and 82± 7% in the cadaveric group.

**Table 2:** Key factors secreted by bone-derived MSCs and their roles in corneal transparency.

Factor	Molecular Mechanism	Clinical Implications
Hepatocyte growth factor (HGF)	Anti-fibrotic, decreases the expression of TGF-β and α-SMA; suppresses TNF-α, MIP-1, IL-6, and CD40.	Reduced corneal opacity and restored transparency with topical application in animal studies.
Soluble fms-like tyrosine kinase-1 (sFLT-1)	Anti-angiogenic, sequesters VEGF ligands, reduces VEGF receptor activation.	Unique among corneal MSCs, sFLT-1 reduces angiogenesis and supports corneal transparency in vitro and animal models.
Pigment epithelium-derived factor (PEDF)	Inhibits VEGF signaling via VEGFR-2 binding, γ-secretase cleavage, and VEGFR-1 phosphorylation alteration.	Inhibits corneal angiogenesis and maintains transparency through multiple pathways.

## Data Availability

Not applicable.
